# Tank fouling community enhances coral microfragment growth

**DOI:** 10.7717/peerj.15723

**Published:** 2023-08-07

**Authors:** Christopher Page, Riley Perry, Claire VA Lager, Jonathan Daly, Jessica Bouwmeester, E. Michael Henley, Mary Hagedorn

**Affiliations:** 1 Smithsonian National Zoo and Conservation Biology Institute, Washington, D.C., United States of America; 2 Hawaii Institute of Marine Biology, Kaneohe, HI, United States of America

**Keywords:** Coral, Coral husbandry, Porites compressa, Restoration, Fouling community

## Abstract

Anthropogenic stressors threaten reefs worldwide and natural *in situ* coral reproduction may be inadequate to meet this challenge. Land-based culture can provide increased coral growth, especially with microfragments. We tested whether culture methods using different algal fouling communities could improve the growth and health metrics of microfragments of the Hawaiian coral, *Porites compressa.* Culture method fouling communities were: (1) similar to a reef environment (Mini Reef); (2) clean tanks managed to promote crustose coralline algae (Clean Start); and (3) tanks curated beforehand with poorly-competing algae (Green Film) assessed in winter and summer months. The Green Film method during the winter produced the fastest microfragment mean growth at 28 days until the first row of new polyps developed, and also the highest tank and plate metric health scores. Time efficient, standardized methods for land-based culture designed to maximize growth and production of coral fragments will contribute considerably to the success of large-scale restoration efforts.

## Introduction

Coral reefs worldwide are dying due to widespread local and global anthropogenic stressors (Hughes et al., 2003; Richmond, Tisthammer & Spies, 2018). This is occurring so rapidly that many coral species may face severe extinction risk by 2100 (Hoegh-Guldberg et al., 2017; Logan et al., 2021). In light of this reef crisis, scalable mechanisms to hasten the recovery of impacted reefs and the adaptation of corals to global stressors have been proposed (National Academies of Sciences E and Medicine, 2019). However, focusing on *in situ* coral propagation strategies alone may be inadequate to meet this challenge, whereas land-based nurseries may significantly augment these restoration needs (Knapp et al., 2022; O’Neil, 2015).

Recent studies with land-based culture techniques have led to increased growth and survival of juvenile coral (Forsman et al., 2015; Hagedorn et al., 2021; Page, Muller & Vaughan, 2018), and have been proposed to augment *in situ* processes *via* large-scale restoration projects such as Mission: Iconic Reefs in Florida (USA) and the Reef Restoration and Adaptation Program in Australia. Despite these successes, outcomes between facilities are often variable due in large part to differing coral culture conditions (Hagedorn et al., 2021; Knapp et al., 2022). This variable performance will greatly impact the utility and scale of land-based culture efforts if prolific, replicable, and transferrable techniques cannot be identified and implemented.

Algal fouling can be a substantial source of competition and mortality for cultured corals. Algae compete with corals *via* direct overgrowth, allelopathy, and by facilitating microbe proliferation (Kuffner & Paul, 2004; Vermeij et al., 2009). Generally, to combat these effects aquarists aim to grow coral by creating a mature reef mesocosm or Mini Reef (*e.g.*, Craggs et al., 2019). Though successful, each Mini Reef system can produce a unique mixture of noxious and benign phototrophic fouling organisms over time, making them difficult to study and replicate. We hypothesized that quickly establishing a simple, poorly-competing, fouling community during coral culture would lead to increased microfragment growth. To test this hypothesis, we used uniformly-sized microfragments from the Hawaiian coral, *Porites compressa*, and compared their growth and health with identified fouling metrics over 15 months using three sequential culture methods: (1) a mini reef mesocosm (Mini Reef); (2) clean tanks managed to promote Crustose Coralline Algae (CCA); and (3) tanks curated beforehand with poorly-competing green algae (Green Film). The results of this study can be used to improve the replicability of land-based coral culture techniques.

## Methods

### Collection of coral

Seventy *Porites compressa* colonies (∼15 cm) were collected from reefs in Kāne’ohe Bay, O’ahu, HI (USA) in accordance with Special Activity Permit numbers 2020-25, 2021-33, and 2022-22 from the Hawai’i Department of Land and Natural Resources. Care was taken to collect colonies at least 15 m apart at different locations and on various reefs throughout Kāne’ohe Bay to avoid collecting clones of the same genotype.

### General care of coral, microfragmentation, and husbandry

**Figure 1:**
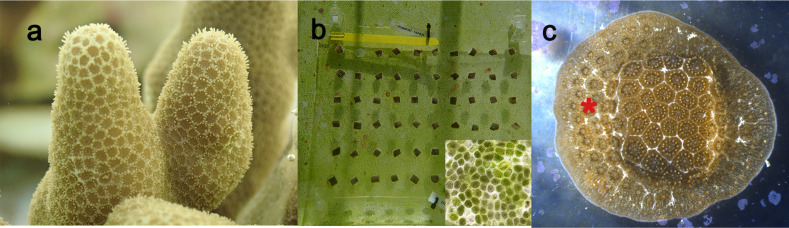
Images of *Porites compressa.* used in these experiments. (A) Colonies of *P. compressa* before cutting. (B) The microfragment array after cutting and gluing the 0.75 × one cm pieces of *P. compressa* onto a plastic sheet clipped to a plexiglass plate for support. The conspicuous green film algae showing through the plexiglass plate. Inset shows details of the phytoplankton cells (∼10 µm in diameter) that constitute the green film. (C) A growing microfragment that has three to four rows of new polyps (*) sheeting out around the cut microfragment. We defined the onset of growth as the date when one ring of new polyps surrounds the microfragment.

These methods were developed and used sequentially as part of a parallel microfragment experiment that required rapid and replicable tissue growth. The general care of coral, process of microfragmentation, and establishing each of the three culture methods can be found in Supplemental Methods. Over time, 70 coral colonies were each cut into 30 uniformly-sized microfragments (1.0 × 0.75 cm) and glued onto a plastic sheet supported by a plexiglass plate (Fig. 1). The sheet allowed for certain microfragments to be harvested for the parallel experiment, as needed. Microfragments were then grown in our husbandry system using three sequential culture methods where the fouling communities were: (1) Mini Reef: a reef mesocosm culture technique used by many laboratories and aquaria; (2) Clean Start: clean tanks managed to promote CCA settlement as described in Hagedorn et al. (2021); and (3) Green Film: tanks curated beforehand with a poorly-competing green algae—a new culture method devised for this project (see Fig. 1B for an image of typical fouling community in the tanks).

In total, we had five tanks that were used for these experiments. One was used solely for the Mini Reef for 4 months, while the other four tanks were used during the Clean Start and the Green Film methods for approximately 1 year. These four tanks were cycled 21 times throughout the remaining months whereby they were continuously setup and torn down to maintain the correct fouling environment of similar age and constituency amongst the tanks. Foulers, consisting of mostly CCA, were targeted for the Clean Start method and mostly green film fouling microalgae were targeted for the Green Film method. These methods are summarized in Table 1.

**Table 1:** Summary culture methods. Methods were implemented sequentially 15 months in 230–460 L culture tanks. For each, tanks were continuously setup and torn down (cycled) to maintain fouling environments of similar age and constituency between tanks.

Husbandry method	Time frame	# Months tested	# Tank replicates /method	# Genotypes	Maintenance hours /tank/day
Mini Reef	Feb 13–May 27, 2020	4	1	3	0.25–0.50
Clean Start	Apr 1–Oct 5, 2020	7	6	17	1.0–4.0
Green Film Summer	Sep 11-Dec 21, 2020; Apr 1–Sep 13, 2021	8.5	9	29	0.25–1.0
Green Film Winter	Dec 4, 2020–Apr 26, 2021	5	6	21	0.25–1.0

### Microfragment growth rate and qualitative metrics

#### Microfragment growth rate

For this study, the onset of new polyps signaled that the coral had healed, resumed calcification, and was in a healthy, robust growth phase. Specifically, this was when the coral fragment had added 0.5 to 1 new row of polyps. To understand how microfragment growth differed amongst the three culture methods, for each sampled microfragment the days from the microfragment cut date until it produced the first ring of new polyps was recorded and compared. The number of genotypes for each treatment for these studies were as follows: Mini Reef: *n* = 3 genets; Clean Start: *n* = 17 genets; Green Film Summer: *n* = 29 genets; and Green Film Winter: *n* = 21 genets.

The parallel experiment required fast microfragment growth, but the necessary parameters that might support this growth were unknown. We suspected that the fouling community might play an important role. Therefore, when we harvested a group of microfragments on a particular day from a specific genotype for the parallel experiment, we assessed the fouling community in the tank and on the plate supporting the microfragments using specific metrics that assessed CCA death, CCA recruitment and nuisance algae coverage. More CCA and less nuisance algae yielded a higher overall health score of the tank and microfragment plate. When we harvested the microfragments for the parallel experiment, we made note of the state of the health of all fragments in the selected genotype. This was a group metric that examined polyp extension, paling of the tissue and new tissue growth across all microfragments in the genotype. Higher overall metrics indicated a healthier overall genotype. These methods are detailed below.

#### Tank and plate scores and microfragment health score

In addition to growth, we developed a suite of qualitative metrics to understand the health of the tank, the plate and the microfragments (that had achieved 1 row of new polyps) in relationship to fouling. During the Clean Start and Green Film methods qualitative metrics composed of three categories were assessed: (1) CCA health: whether CCA was growing or dying in the tank; (2) CCA recruitment: the ratio of new CCA growing in the tank compared to other competing algae; and (3) nuisance algae coverage: how much and what type of algae was growing throughout the tank (see Table S1 for scoring criteria). Fouling was visually assessed on all surfaces of the tank including any added substrates (*i.e.,* heaters, air stones, snail cages, etc.) at the meter scale. These values were then totaled to determine a score for each metric. The higher the score, the healthier the tank or plate was considered to be. The Clean Start method was only used during the summer as it became supplanted by the Green Film method. Data collected during the green film method was divided into values collected during winter months (Dec 1 to Mar 30) and all other months to determine if season effected outcome. As the qualitative metrics were developed in the early stages of the Clean Start method they were not available for application to the earlier Mini Reef approach.

Health metrics for microfragments were assessed and compared, because each microfragment experienced differing conditions based on their position among and within culture tanks, using three metrics: (1) polyp extension: the percentage of microfragments on the plate that had their polyps extended; (2) change in coloration/tissue recession: the number of microfragments that had paled or had tissue recession; and (3) new tissue integrity: the thickness and color of the tissue surrounding the microfragment and its rate of advancement (see Table S2). The higher the score, the healthier the microfragment was considered to be.

### Statistical methods

To determine the difference in microfragment growth rates in response to each of the three culture methods, the number of growth days were first converted to categorical variables, in 14-day groupings, and analyzed with a repeated ordinal regression with a cumulative link mixed model (CLMM), with culture method as fixed variable and genotype as random variable. To determine the difference in fouling and health metrics, the scores were analyzed with a repeated ordinal regression with CLMM, with culture method as fixed variable and genotype as random variable. Significant results were followed with a estimated marginal means (EMmeans) posthoc test with Tukey adjustments for multiple comparisons. Proportional odds assumptions were verified using tests of nominal effects and tests of scaling effects. All statistical analyses were performed in R (R Core Team, 2019), with the packages emmeans (Lenth, 2022), ordinal (Christensen, 2022), multcomp (Hothorn, Bretz & Westfall, 2008), and rcompanion (Mangiafico, 2022). All error bars in the figures represent the standard error of the mean, unless otherwise stated.

## Results

**Figure 2:**
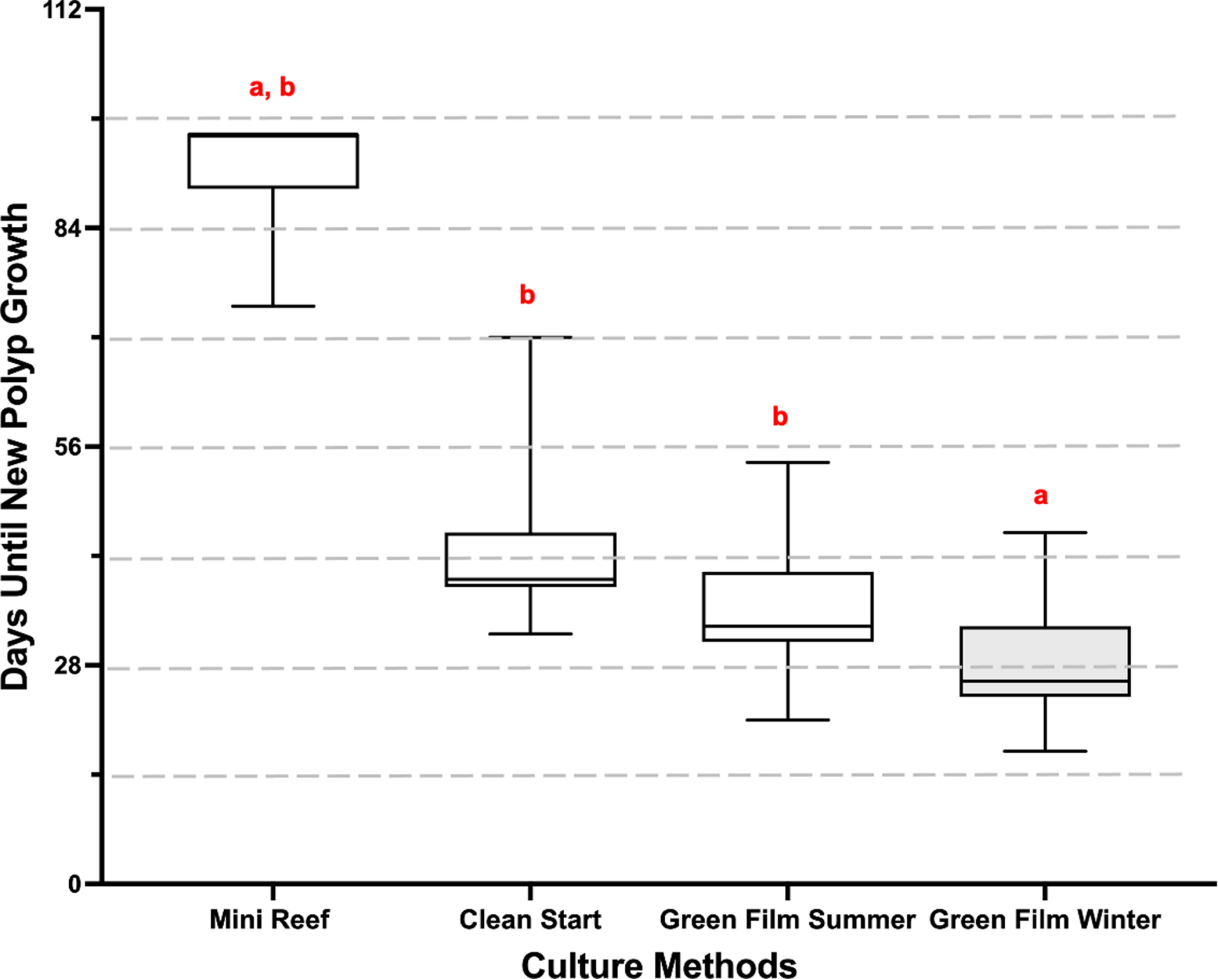
Mean growth rates of the *P. compressa* microfragments under different culture methods. The time from microfragmentation until the first row of new polyps developed was measured for each culture method and season. The data were assessed and represented by their number of growth days. Then, the number of growth days were converted to categorical variables, in 14-day groupings (indicated by dashed grey line), and analyzed with a repeated ordinal regression with cumulative link mixed model (CLMM), with culture method as fixed variable and genotype as random variable (${\chi }_{(3)}^{2}=77.3$, *p* < 2*x*10^−16^). In the graph a box represents the central 50% of the data distribution, the bar in the box represents the median, the whiskers represent the remaining 50% data spread, and different letters above the boxes indicate significant differences in the treatments. The Green Film method overall produced the most rapid growth especially during winter months (28.3 ± 0.5 days).

Time to first growth was defined as the total number of days from initial cut to generation of the first ring of polyps, which we then converted to 2-week categories (Figs. 1 and 2). The culture method had a significant effect on microfragment growth (repeated ordinal regression with CLMM, ${\chi }_{\mathrm{(3)}}^{2}=77.3$, *p* < 2 × 10^−16^). The Green Film method overall produced the most rapid growth especially during winter months (28.3 ± 0.5 days; Fig. 2), and was significantly shorter than the Clean Start method and the summer Green Film method (EMmeans posthoc test). The Mini Reef method yielded the slowest growth (91.9 ± 3.1 days) although due to small sample size no significant difference was detected between the Mini Reef method and the other culture methods (EMmeans posthoc test).

**Figure 3:**
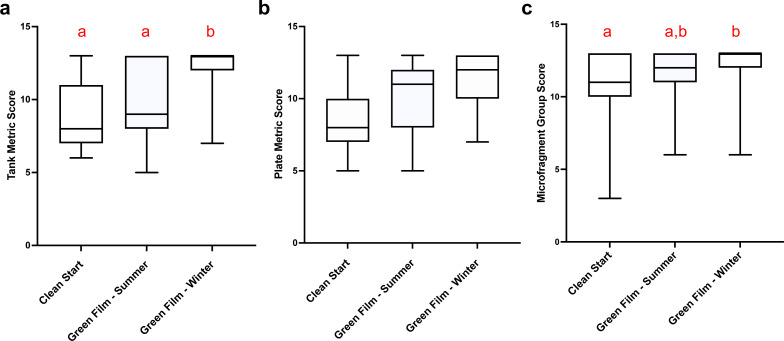
Fouling and health metrics *versus* culture methods. The tanks (A) and plates (B) in various tanks in different culture methods were scored by assessing tank fouling metrics; (1) CCA health; (2) CCA recruitment; and (3) nuisance algae coverage. (C) The microfragments in these tanks were scored by assessing: (1) polyp extension; (2) change in coloration/ tissue recession; and (3) new tissue integrity. The Green Film Winter had the highest overall mean metric scores producing the most benign fouling and strongest health for the microfragments at that time. The Green Film Summer was second highest and the Clean Start method had the lowest means. Different letters indicate significant differences (*α* = 0.05), as determined by Dunn’s Multiple Comparison *posthoc* test. In the graph a box represents the central 50% of the data distribution, the bar in the box represents the median, the whiskers represent the remaining 50% data spread, and different letters above the boxes indicate significant differences in the treatments. Error bars are SEM.

The Green Film method conducted during the winter produced the highest mean tank health scores (repeated ordinal regression with CLMM, ${\chi }_{\mathrm{(2)}}^{2}=23.5$, *p* = 8 × 10^−6^, EMmeans posthoc test, Fig. 3A) and the highest mean microfragment health scores (repeated ordinal regression with CLMM, ${\chi }_{\mathrm{(2)}}^{2}=7.0$, *p* = 0.03, EMmeans posthoc test, Fig. 3C). The culture method did not have any effect on the mean plate health scores (repeated ordinal regression with CLMM, ${\chi }_{\mathrm{(2)}}^{2}=3.6$, *p* = 0.169, Fig. 3B). See Tables S1 and S2 for metrics definitions.

The Green Film method required managing the formation of an algal film throughout the tank to occupy vacant substrate that might otherwise be colonized by noxious foulers prior to placing microfragments into the tank. This method resulted in healthier CCA, an important coral settlement inducer, and less nuisance algae present on tank surfaces when microfragments were sampled for this study. Specifically, when compared to the Clean Start method the Green Film method in winter resulted in ∼50% higher CCA health and recruitment scores and a concomitant ∼13% increase in nuisance algae scores (higher scores mean less nuisance algae).

## Discussion

Biofilms, like the green alga studied here, play a critical role in the growth and development of coral reefs and have been shown to influence coral recruitment and survival as well as inhibit algal settlement. A biofilm of bacteria and microalgae achieved 41% higher settlement of *Acropora micropthalma* larvae (Webster et al., 2004). Additionally, strains of *Pseudoalteromonas* sp. isolated from Crustose Coraline Algae induced metamorphosis in many other species of coral larvae (Sneed et al., 2014; Tebben et al., 2011). Conversely, the cyanobacteria *Hormothamnion enteromorphoides* inhibited settlement in *Pseudidiploria strigosa* and *Caldora penicillata* inhibited both settlement and survival in *Acropora palmata* larvae (Ritson-Williams, Arnold & Paul, 2020). Biofilms can also inhibit algal settlement (Gomez-Lemos & Diaz-Pulido, 2017). These authors demonstrated that both Crustose Coraline Algae and microbial biofilms significantly reduced settlement of the seaweed *Padina boergesenii* through allelopathic action and microbial inhibition. Diatom settlement and germination of *Ulva lactuca* was inhibited by culturing a biofilm composed of a *Alteromonas sp* (Silva-Aciares & Riquelme, 2008). It is hypothesized that the green film algae grown in this study may influence coral health and rate of tissue expansion in coral microfragments by reducing more deleterious competition.

The higher performance with growth and fouling metrics of the Green Film Winter compared to the Green Film Summer may have been facilitated by a number of factors. Although the tanks generally had consistent biophysical parameters throughout the year, the reduced temperature of the incoming seawater prior to system heating and reduced irradiance during the winter months may have slowed the colonization of nuisance algae, allowing for its more effective eradication using gastropod grazers.

In this study, all fouling control methods were effective at preventing filamentous algae overgrowing fragments, but cyanobacteria was not equally controlled. Cyanobacteria was present in substantial quantities in the Mini Reef and constantly in need of control in the Clean Start method using grazers and periodic remediation. In contrast, cyanobacteria was nearly absent from the Green Film tanks. Noxious phototrophs, such as cyanobacteria, are suspected to engage in allelopathy and elicit poor microbial ecology (Kuffner & Paul, 2004; Vermeij et al., 2009). Kuffner & Paul (2004) found that the presence of the cyanobacteria *Lyngbya majuscula* in coral settlement chambers significantly decreased survival of *Acropora surculosa* larvae and recruitment on unoccupied substrate in *Pocillopora damicornis* (Kuffner & Paul, 2004). Similar deleterious effects may have occurred in the Mini Reef and Clean Start tanks, as well.

**Table 2:** Advantages and disadvantages of each culture method for restoration.

Culture Method	Advantages	Disadvantages
*Mini Reef*	• Familiar strategy • Approximates fouling of a reef • Low maintenance time once established (0.25–0.50 h/tank/day) • Minimizes algal overgrowth of microfragments • No tank restarts needed	• Waiting required—lengthy and variable establishment times (1 + yr) • Slowest grow out time achieved here • Cyanobacteria potentially problematic • Harbors an irreplicable mixture of foulers, potentially resulting in a tank effect
*Clean Start*	• Easy to start, no waiting • Reduced subset of foulers to manage due to early settlement dynamics • Fouling actively managed • Establishes CCA quickly • Fast grow-out time	• High maintenance time (1–4 h/tank/day) • Threat of nuisance algae bloom is high throughout the tank’s life • Restart required in 4–6 months to avoid mini reef conditions
*Green Film*	• Preselects for benign green algae • Low maintenance time (0.25–1 hour/tank/day) • Simple, replicable fouler management • Fastest grow-out time • Nuisance algae blooms are infrequent	• Two-week waiting period for film to develop • Restart required in 4–6 months to avoid mini reef conditions

Despite its low sample size, we nonetheless felt it important to include the Mini Reef method because the mean growth values for the Clean Start and Green Film methods were substantially different from the Mini Reef. This suggests that this commonly employed approach may not always be best for growing juvenile coral quickly. Additionally, its inclusion frames this study in a context familiar to coral culturists and will aid discussions aimed at scaling this work for restoration. Here we highlight two major shortcomings to this Mini Reef strategy: (1) highly variable outcomes between separate mesocosms (Petersen et al., 2006); and (2) the significant time investment required to produce a late-stage reef environment. This study proposes some potential alternate culture methods and provides enough context to weigh the pros and cons of utilizing each option, providing a decision-making tool that is sorely needed (Table 2). The Green Film method reduced the mean growth time and was more replicable because it: (1) had an easy and relatively short startup procedure; (2) created a standardized fouling community; and (3) used active grazer management.

Although tank effects can be a concern in land-based studies, if a tank effect was present in this study, it would have been distributed across all the treatments and data (except the Mini Reef), because as we set up a new treatment we rotated the treatments from tank-to-tank in our flow through water system throughout the year. For this reason, we did not include tank effect as a factor in analyzing these experiments.

The algae observed here, is likely a benign microalga that keeps more noxious agents reduced in tanks, allowing the coral to grow more rapidly. We believe that similar benign biofilms occur in other areas of the world, such as the Caribbean (C. Page pers. observation) and could easily be incorporated into future husbandry work. Additionally, because of the rapid growth potential of the green film algae compared to CCA witnessed here, cultivated strains may serve as useful inoculum to rapidly establish good growing conditions in new tanks. Continued investigation is needed to identify the genus of the alga cultivated here as well as to identify similar species in other parts of the world.

Finally, it is important to acknowledge that this work was accomplished on a single Hawaiian endemic coral and therefore may not be representative of how all species will perform in these environments. However, if these methods prove useful in other species, we suggest that the Green Film method may be a strategy to improve land based coral culture outcomes for use in restoration and experimentation.

##  Supplemental Information

10.7717/peerj.15723/supp-1 Supplemental Information 1 Supplemental methods and data

10.7717/peerj.15723/supp-2 Data S1 Raw Data

## Additional Information and Declarations

### Data Availability

The following information was supplied regarding data availability: The raw data is available in the Supplemental Files.

## References

[ref-1] Christensen RHB (2022). https://cran.r-project.org/package=ordinal.

[ref-2] Craggs J, Guest J, Bulling M, Sweet M (2019). *Ex situ* co culturing of the sea urchin, Mespilia globulus and the coral Acropora millepora enhances early post-settlement survivorship. Scientific Reports.

[ref-3] Forsman ZH, Page CA, Toonen RJ, Vaughan D (2015). Growing coral larger and faster: micro-colony-fusion as a strategy for accelerating coral cover. PeerJ.

[ref-4] Gomez-Lemos LA, Diaz-Pulido G (2017). Crustose coralline algae and associated microbial biofilms deter seaweed settlement on coral reefs. Coral Reefs.

[ref-5] Hagedorn M, Page CA, O’Neil KL, Flores DM, Tichy L, Conn T, Chamberland VF, Lager C, Zuchowicz N, Lohr K, Blackburn H, Vardi T, Moore J, Moore T, Baums IB, Vermeij MJA, Marhaver KL (2021). Assisted gene flow using cryopreserved sperm in critically endangered coral. Proceedings of the National Academy of Sciences of the United States of America.

[ref-6] Hoegh-Guldberg O, Poloczanska ES, Skirving W, Dove S (2017). Coral reef ecosystems under climate change and ocean acidification. Frontiers in Marine Science.

[ref-7] Hothorn T, Bretz F, Westfall P (2008). Simultaneous inference in general parametric models. Biometrical Journal.

[ref-8] Hughes TP, Baird AH, Bellwood DR, Card M, Connolly SR, Folke C, Grosberg R, Hoegh-Guldberg O, Jackson JBC, Kleypas J, Lough JM, Marshall P, Nyström M, Palumbi SR, Pandolfi JM, Rosen B, Roughgarden J (2003). Climate change, human impacts, and the resilience of coral reefs. Science.

[ref-9] Knapp ISS, Forsman ZH, Greene A, Johnston EC, Bardin CE, Chan N, Wolke C, Gulko D, Toonen RJ (2022). Coral micro-fragmentation assays for optimizing active reef restoration efforts. PeerJ.

[ref-10] Kuffner IB, Paul VJ (2004). Effects of the benthic cyanobacterium Lyngbya majuscula on larval recruitment of the reef corals Acropora surculosa and Pocillopora damicornis. Coral Reefs.

[ref-11] Lenth R (2022).

[ref-12] National Academies of Sciences E and Medicine (2019). A research review of interventions to increase the persistence and resilience of coral reefs.

[ref-13] Logan CA, Dunne JP, Ryan JS, Baskett ML, Donner SD (2021). Quantifying global potential for coral evolutionary response to climate change. Nature Climate Change.

[ref-14] Mangiafico S (2022). https://cran.r-project.org/package=rcompanion.

[ref-15] O’Neil KL (2015). Land-based coral nurseries: a valuable tool for production and transplantation of *Acropora cervicornis*. Master’s thesis.

[ref-16] Page C, Muller E, Vaughan D (2018). Microfragmenting for the successful restoration of slow growing massive corals. Ecological Engineering.

[ref-17] Petersen D, Laterveer M, Bergen D, Hatta M, Hebbinghaus R, Janse M, Jones R, Richter U, Ziegler T, Visser G, Schuhmacher H (2006). The application of sexual coral recruits for sustainable management of *ex situ* populations in public aquariums—SECORE-Project. Aquatic Conservation: Marine and Freshwater Ecosystems.

[ref-18] R Core Team (2019). https://www.R-project.org.

[ref-19] Richmond RH, Tisthammer KH, Spies NP (2018). The effects of anthropogenic stressors on reproduction and recruitment of corals and reef organisms. Frontiers in Marine Science.

[ref-20] Ritson-Williams R, Arnold SN, Paul VJ (2020). The impact of macroalgae and cyanobacteria on larval survival and settlement of the scleractinian corals Acropora palmata, A. cervicornis and Pseudodiploria strigosa. Marine Biology.

[ref-21] Silva-Aciares FR, Riquelme C (2008). Inhibition of attachment of some fouling diatoms and settlement of Ulva lactuca zoospores by film-forming bacterium and their extracellular products isolated from biofouled substrata in Northern Chile. Electronic Journal of Biotechnology.

[ref-22] Sneed JM, Sharp KH, Ritchie KB, Paul VJ (2014). The chemical cue tetrabromopyrrole from a biofilm bacterium induces settlement of multiple Caribbean corals. Proceedings of the Royal Society B: Biological Sciences.

[ref-23] Tebben J, Tapiolas DM, Motti CA, Abrego D, Negri AP, Blackall LL, Steinberg PD, Harder T (2011). Induction of larval metamorphosis of the coral Acropora millepora by tetrabromopyrrole isolated from a Pseudoalteromonas bacterium. PLOS ONE.

[ref-24] Vermeij MJA, Smith JE, Smith CM, Vega Thurber R, Sandin SA (2009). Survival and settlement success of coral planulae: independent and synergistic effects of macroalgae and microbes. Oecologia.

[ref-25] Webster NS, Smith LD, Heyward AJ, Watts JE, Webb RI, Blackall LL, Negri AP (2004). Metamorphosis of a scleractinian coral in response to microbial biofilms. Applied and Environmental Microbiology.

